# Challenges of after-hours pediatric imaging

**DOI:** 10.1007/s00247-025-06473-2

**Published:** 2025-11-28

**Authors:** Michael R Aquino, Jignesh Shah, Summer L Kaplan

**Affiliations:** 1 Imaging Institute, Cleveland Clinic, 9500 Euclid Ave, Cleveland, OH 44195 United States; 2 Cleveland Clinic Lerner College of Medicine, Cleveland, United States; 3 Texas Children’s Hospital, Houston, United States; 4 Baylor College of Medicine, Houston, United States; 5 Children’s Hospital of Philadelphia, Philadelphia, United States; 6 University of Pennsylvania, Philadelphia, United States

**Keywords:** Emergency, Shift-work schedule, Medical errors, Sleep deprivation, Pediatric, Radiology, Radiologists, Workforce, Circadian rhythms

## Abstract

The after-hours period presents unique challenges for pediatric radiologists. These stem from the higher percentage of high acuity/emergent studies, limited staffing, and the adverse effects of non-traditional hours on radiologists’ health and performance. This article describes the current landscape of after-hours pediatric radiology coverage, workflow, and staffing challenges, and reviews the impact that disruptions in circadian rhythm can have on health and performance.

## Introduction

There is growing demand for specialized interpretation of pediatric imaging 24 h a day, 7 days a week (24/7) [1]. This is underscored by the finding that the major discrepancy rate between interpretations by non-fellowship-trained attending radiologists and attending pediatric radiologists has been reported as high as 21.7% [2]. The demand is further driven by patient safety concerns and quality metrics such as turnaround time and emergency department length of stay in the setting of increased patient volumes and imaging utilization [1]. Cross-sectional imaging (ultrasound, computed tomography, and magnetic resonance imaging) use in pediatric emergency departments increased in the USA from 6.4% of patient encounters in 2009 to 8.7% in 2018. This increase was driven by the non-radiating modalities, ultrasonography and MRI. Ultrasonography use increased from 2.5% to 5.8% of emergency department encounters, and MRI use doubled from 0.3% to 0.6% of emergency department encounters during the same period [3]. In contrast, CT has driven increases in imaging utilization at non-pediatric general community and academic centers based on national survey data. For instance, CT utilization per 1000 Medicare fee-for-service enrollees increased 153% from 2004 to 2016 [4]. These increases also result in a significant rise in the number of images to be reviewed.

## After-hours coverage models and staffing challenges

The after-hours period is any time outside of regular business hours. The specific hours used to define the period are variable in the literature, but a commonly used time period is from 5 PM to 8 AM [5, 6]. The challenge of providing after-hours pediatric radiology service is in maintaining a consistent quality of care despite limited resources, while minimizing disruption to daytime staffing, and ensuring financial feasibility, and sustainability for both the pediatric radiologist and the institution. There is no one-size-fits-all model. The model must align with group culture, staffing, and the goals of the institution. The need for on-call on-site availability for emergent fluoroscopic procedures is another key consideration. As a result, different institutions use a variety of methods to provide after-hours coverage (Table 1).

**Table 1:** After-hours coverage models in pediatric radiology

Model	Description	Advantages	Challenges
Trainee-only coverage	Preliminary interpretations provided by residents/fellows	Financially efficient; provides autonomy to trainees	Risk of discrepancies; medico-legal concerns
Rotating staff shifts	Daytime staff rotate through overnight/evening schedules	Familiarity with both day and night workflows; continuity of care	Scheduling complexity; risk of burnout; may not suit subspecialists and limit ability to maintain subspecialty practice
Teleradiology	Remote interpretation by contracted radiologists	Expands subspecialty access; scalable; flexible use for surges	Dependent on IT infrastructure; cannot replace on-site procedures; integration challenges
Emergency radiology team	Dedicated pediatric radiologists covering nights	Simplifies scheduling; supports recruitment	Costly to implement; requires broad skill-set; feasible mainly in larger centers

Recent surveys provide insight into the current landscape of after-hours pediatric imaging coverage [2, 7–9]. The majority of institutions in these surveys provide interpretations of pediatric imaging 24/7. Many still rely heavily on trainee-only coverage and preliminary reports. Attending coverage, however, is becoming more common via teleradiology, rotation of existing staff into after-hours shifts, or through the creation of emergency radiology teams. Similar models of coverage are utilized at general community and academic centers [7].

### Trainee coverage

Trainee-only coverage with preliminary interpretations for at least a part of the after-hours period was reported by 38–43% of respondent institutions [8, 9]. Financially, a trainee-only coverage model is beneficial as it utilizes existing resources. The drawback is the risk of errors, but advocates of trainee-only coverage note that discrepancy rates between trainee and attending interpretations are very low, with clinically significant discrepancies even lower [10]. Notably, for pediatric trauma studies, the discrepancy rate between radiology residents and non-fellowship-trained teleradiologists has been reported to be comparable [10]. Proponents of 24/7 attending coverage, however, emphasize limited pediatric radiology training during residency with potential for higher discrepancy rates for pediatric cases, and the potential for clinically significant discrepancies to be life-threatening, despite being rare [10, 11]. For instance, trainee misinterpretations of pediatric neuroimaging studies were identified in 4.1% of studies and 0.17% of these errors were potentially life-threatening [10]. Consequently, the financial advantages of trainee-only coverage must be weighed against risk and the potential litigation for a perceived breach in the standard of care [1].

The impact of 24/7 attending coverage on trainee learning is also debated. While autonomy is a valuable experience, direct supervision may enhance education. Evidence supports both views [12–14]. Ultimately, institutions must decide how their solutions for after-hours coverage will balance the need for quality patient care and trainee education.

### Rotating shift-work

Existing staff can cover after-hours using a rotating shift-work model. In this model, the 24-h day is divided into sequential and/or overlapping blocks of time that existing staff rotate through to ensure around-the-clock coverage. Thirty-nine percent of respondent institutions reported using this model of coverage [8]. Use of existing staff ensures radiologist familiarity with both the daytime and overnight practices but complicates scheduling. Balancing days, evenings, overnights, post call time, vacation time, and conference time is challenging, especially in smaller groups and may limit the ability to maintain a subspecialty practice. Additionally, some staff may not be well-suited for overnight work due to the physiological challenges or required generalist skills.

### Teleradiology

Teleradiology can suit a variety of group sizes and is utilized by 20–30% of institutions [8, 9]. It offers the opportunity to provide subspecialized interpretation of imaging studies after-hours, even for smaller groups and geographically remote locations. The services provided may vary based on the needs of the contracting institution. For instance, institutions may have the option of selecting preliminary or final reports, and the ability to utilize the teleradiology service in times of volume surges or to help clear significant case backlogs. However, institutions still require that a pediatric radiologist be on-call for on-site fluoroscopic procedures and internet or IT downtime.

It is important to note that the American College of Radiology offers best practice guidelines in a published white paper on teleradiology practice from 2013. In it, they recommend that (1) teleradiologists have complete access to the electronic medical record and prior studies, (2) communication between the teleradiologist and referring physician be readily and bidirectionally available, and (3) that teleradiologists be incorporated into the local operations related to safety and quality within the radiology practice, including peer review and peer learning activities [15].

### Emergency radiology team

Emergency radiology teams were used by 25% of respondent institutions and are composed of pediatric radiologists that predominantly or exclusively work overnight [8]. These radiologists are typically proficient in a broad range of studies including fluoroscopy and neuroradiology and have familiarity with emergency and trauma imaging protocols. Emergency radiologists may develop strong working relationships with staff from other departments such as emergency medicine, surgery, and pediatrics that may similarly work many after-hours shifts. This model also simplifies scheduling, avoids disruption to daytime staffing, and may support staff recruitment and retention. The cost of creating such a team, however, may be prohibitive particularly for smaller institutions [1].

### Scheduling

There are no formal evidence-based recommendations from radiology societies on structuring after-hours schedules. Broader guidelines published by the American College of Occupational and Environmental Medicine recommend limiting shift duration and the number of consecutive night shifts to reduce the risk of errors and adverse health outcomes but do not specify a maximum duration or consecutive number of shifts [16]. More detailed recommendations from other fields suggest schedules have (1) ≤3 consecutive night shifts; (2) shift intervals of ≥11 h; and (3) ≤9 h shift duration [17].

In practice, after-hours schedules are highly variable in pediatric centers as well as in general community hospitals and academic centers [7–9, 18]. This variability reflects the need for each group to develop its service based on its unique combination of resources, workforce, objectives, and needs of the referring physicians and patient population. Discussion with group members, stakeholders from other departments, and administration is an important step to ensure the service model aligns with the needs and culture of the institution. Importantly, the different models of coverage are not mutually exclusive and institutions may utilize multiple in combination to provide 24/7 imaging services.

Despite the variability in most after-hours schedules, three points are important to highlight (Table 2). First, the most common schedule structure used in the pediatric emergency radiology team model is 7 consecutive overnight shifts followed by 14 days off with typical shift start and end times of 9 PM or 10 PM and 7 AM or 8 AM, respectively [8, 19]. Radiologists on a 1 week on, 2 weeks off schedule were significantly more likely to perceive being able to stay in their position for >5 years compared to those on a 1 week on, 1 week off schedule (Fisher’s exact test *P*=0.015). Radiologists on a 1 week on, 2 weeks off schedule also had the highest proportion of respondents who had actual duration in an after-hours position of >5 years (6/23, 26.1%) [19]. Qualitative responses from the same survey included comments that a schedule of 1 week on followed by 1 week off did not provide sufficient time for recovery and resulted in burnout [19].

**Table 2:** After-hours scheduling considerations

Model (schedule pattern)	Scheduling	Staffing	Traits/considerations
“1 week on/2 weeks off”	7 consecutive overnight shifts followed by 14 days off	Requires a larger pool of radiologists to cover long off-service intervals	Better long-term retention and sustainability for staff; provides ample recovery time between blocks of nights
“1 week on/1 week off”	7 consecutive overnight shifts followed by 7 days off	At least two rotating radiologists alternating weeks (smaller overall workforce than 7/14 model)	Associated with higher burnout and insufficient recovery time for individuals; less sustainable long-term
Rotating shifts	Day → Evening → Overnight in rapid succession (usually ≤4 consecutive night shifts)	All faculty share night duty in rotation; scheduling is complex, requiring flexibility from the entire team	Forward (clockwise) rotation causes less circadian disruption than backward rotations; may disrupt subspecialty practice due to frequent schedule changes; self-selection of after-hours schedule improves job satisfaction and work-life balance

Second, meta-analyses have shown that the direction and speed of rotation in a rotating shift-work model are important variables. Specifically, forward shift-work rotation (day to evening to overnight) and fast rotation (3 or 4 consecutive night shifts compared to 6 or 7) may be less detrimental to the health and work-life balance of workers [20].

Third, evidence suggests that after-hours workers who are provided the opportunity to play an active role in the creation of their work schedule have increased job satisfaction and improved perception of work-life balance. This can be a useful strategy for retention and recruitment and comes at minimal or no direct cost to the organization [20–22].

### Factors affecting after-hours staffing

The push for 24/7 attending pediatric radiology staffing faces challenges. First, there is a shortage of pediatric radiologists in the workforce and the number of fellowship graduates is not expected to meet the growing need [23, 24]. Second, the challenges of working after-hours shifts may deter radiologists from pursuing such work. Consequently, it becomes imperative for groups to identify individuals with an aptitude for after-hours work.

#### Deterrents to pursuing after-hours work

The majority of people that left their after-hours positions did so within 3 years (67%). Of those who remained, 44% said they did not anticipate continuing more than 5 years [19]. Several factors deter people from pursuing after-hour shift-work positions or limit their longevity in these positions. In survey data, more than half of respondents left their job or reported anticipated limited tenure due to difficulties with changing sleep schedules. Other deterrents identified in survey data included limited advancement opportunities, understaffing, lack of subspecialty support, disruption to personal life, and feelings of isolation [19]. Notably, the effects of after-hours work were reported to extend beyond the professional setting, influencing interpersonal relationships, participation in social activities, and overall quality of life. The association of after-hours shift-work with increased disease risk is also a significant concern. For instance, the number of annual nightshifts worked was significantly associated with the perception that job schedule negatively impacted health in a survey of 327 radiologists (*P*<0.01) [18].

These deterrents highlight why recruitment and retention remain significant challenges in sustaining after-hours pediatric radiology services. Certain individuals, however, may have an affinity and aptitude for after-hours work.

#### Traits that support after-hours tolerance

Survey data and observational studies suggest that certain individual traits may mitigate some of the challenges of working after-hours, contribute to job satisfaction, and support longevity in after-hours positions.

##### Evening chronotype

An individual may have an aptitude to work after-hours shifts if they are better able to tolerate the late hours and circadian shifts. “Morningness” is a measure of alertness in the morning. Studies have shown that people with high scores of morningness have more difficulty adjusting to overnight work. In contrast, people with low scores of morningness, also known as “evening types,” report a better ability to adapt to after-hours shift-work, tolerance for circadian shifts, better after-hours work performance, and higher job satisfaction [25].

##### Younger age

The ability to tolerate shifts in circadian rhythm also diminishes with age which results in increased vulnerability to sleep loss [26]. Thus, younger radiologists may be more resilient to after-hours schedules [27].

##### Hardiness

Hardiness refers to a set of characteristics that enhance resilience to stressful events and has been shown to be inversely related to measures of insomnia, sleepiness, depression, and anxiety in after-hours workers [25].

##### Practice experience

Data on the effect of practice experience on the ability to acclimate to an after-hours position is conflicting. In a survey of 327 radiologists, those with more than 10 years of practice experience who worked 0–100 after-hours shifts per year indeed had decreased odds of feeling overwhelmed compared to less experienced radiologists. However, odds of feeling overwhelmed at work were lower for radiologists who worked 26 weeks or more of after-hours shifts per year compared to those who worked none, regardless of experience level [18]. Authors of this study suggest that these results reflect a self-selection bias in that people who are willing to work a large number of after-hours shifts may have traits that may facilitate acclimation to such work.

A survey of pediatric radiologists with experience in after-hours work supports the suggestion that these positions may be appropriate for pediatric radiologists immediately after or a few years out of fellowship. When people with after-hours experience were surveyed, 67% indicated that they would recommend after-hours positions to fellows. Those who would not recommend after-hours work cautioned that in their opinion the case-mix and professional isolation were not ideal for recent graduates. Despite these concerns, radiologists with less than 1 year of attending experience prior to entering an after-hours position were (1) the largest respondent group (14/44, 32%), (2) had the greatest number and proportion of radiologists that remained in their position for >5 years (5/14, 36%), and (3) and were the most likely to recommend an after-hours position to fellows (chi-square test=10.18, *P*=0.006) [19]. This underscores the importance of active mentorship and sustained opportunities for professional growth and academic engagement to support early-career radiologists in these roles.

##### Skill-set

Those able to tolerate the physiologic stresses of after-hours work may find the required generalist skill-set challenging if their daytime practice is more subspecialty focused. A broad skill-set with proficiency in body, neuro, and musculoskeletal imaging ensures the ability to manage the breadth of cases received from the emergency department, urgent care centers, and inpatient units [3]. Radiologists with in-house work expectations may also require fluoroscopic skills.

While individual traits may mitigate some of the challenges of after-hours work, they cannot fully eliminate the consequences of circadian disruption and sleep deprivation encountered in these roles. An awareness of the effects of after-hours schedules on cognition, performance, and health as well as strategies to manage these is important to ensure that radiologists in these roles are supported appropriately.

## Health and performance of after-hours workers

### Cognitive impairment

Sleep deprivation impairs cognitive function. The range of effects on cognitive performance is broad and the degree of impairment worsens as time on task is increased [28, 29]. Subjects can experience cognitive slowing which, under time pressure, can result in increased errors. Short-term and working memory can be impaired and lead to inefficiencies and errors. Divergent thinking skills can also decline and adversely impact decision-making. Subjects may perseverate, lose situational awareness, and can experience a reduced ability to assimilate new information, assess risk, and consider alternative solutions [29]. Mood changes, irritability, and diminished communication skills can also occur.

An example of the above sleep-related cognitive function impairments in radiologists is demonstrated in a study by Hanna et al. in which radiologists were asked to review 20 bone radiographs (15 positive for fracture and 5 normal) on 2 occasions: at the end of a 9-h daytime shift, and at the end of a 9-h overnight shift [30]. Compared to after daytime shifts, after the overnight shifts, the radiologists had higher markers of fatigue (*P*<0.01), worse diagnostic performance (*P*<0.05), longer total viewing time per case (mean 35.9±25.8 s versus 24.8±16.3 s, *P*<0.0001), and longer mean time to first fixate on a fracture (mean, 14.9±7.02 s versus 11.1±5.7 s, *P*<0.0001). Finally, dwell times (time spent fixated on a region of interest) were longer for decisions that resulted in true positive outcomes (mean 839.34±419.15 ms versus 715.63±410.69 ms, *P*=0.025), and shorter for decisions that resulted in false negative outcomes (mean 547.03±375.64 ms versus 689.30±403.33 ms).

Microsleep, short involuntary sleep lasting a few seconds, is another consequence of sleep deprivation [29]. The subject often does not realize when a microsleep occurs. Compensatory mechanisms may conceal these episodes. Subjects may appear awake with eyes open or ambulate but information is not appropriately processed by the brain. Microsleeps are associated with motor vehicle accidents and may contribute to errors in overnight workers.

### Errors

Multiple studies in various fields including medicine have shown that there is an increased risk of errors and accidents in evening and overnight shifts which predispose individuals to sleep deprivation [28, 29, 31]. A study that reviewed data across multiple industries observed a relative risk of injuries and accidents of 1.3 for night shifts [32]. The relative risk for hospital night shifts was 1.81. Risk was also shown to increase with shift length.

In studies specific to radiologists, researchers found that errors more commonly occur later in the shift (mean 8.97 h into the shift), peaking between 10 and 12 h [33]. Studies have also shown that a greater number of major discrepancies occur in overnight shifts compared to daytime shifts. For radiographs, US, and CT, the major discrepancy rates were 0.13% for daytime shifts and 0.14% for overnight shifts (*P*<0.01). Similar results were found for CTA and MRI studies with major discrepancy rates of 0.15% for daytime shifts, 0.20% for evening shifts, and 0.23% for overnight shifts (daytime versus evening *P*=0.028, daytime versus overnight *P*<0.01) [34].

Some inconsistencies in the after-hours error rates of radiologists, however, exist. In an analysis of peer learning data, Sammer et al. calculated the learning opportunity rates of pediatric radiologists categorized by time of report dictation. The highest rates were found in radiologists working between 5 PM and 10 PM, but the lowest rates were found in radiologists working 10 PM and 7 AM despite this shift averaging the highest volume and second highest wRVU per radiologist-hour. The authors suggest that these findings indicate factors other than time of report, volume, and wRVU contribute to the rate of peer learning opportunities [35].

### Increased disease risk

Another significant deterrent to working after-hours is the increased risk of disease, including cardiovascular disease, gastrointestinal disease, psychiatric disorders, reproductive issues, diabetes, and cancer [36–41]. Physiologic and genetic factors are key contributors to increased disease risk. Behavioral factors that may occur during after-hours work, including adverse changes in eating habits and physical activity, are also important considerations [42, 43].

#### Circadian rhythm and the endocrine system

The circadian rhythm influences endocrine factor levels and target organ sensitivity [44]. For instance, endocrine factors such as cortisol, ghrelin, leptin, vasopressin, and melatonin exhibit predictable oscillations with a 24-h periodicity which aligns physiological functions with the light-dark cycle. Under normal conditions, this alignment promotes efficient organ function through the appropriate anticipation of daytime and nighttime activities. However, in the setting of after-hours work, endocrine activity and physiologic function become desynchronized from behavior and can lead to pathology. Consider the effects of cortisol as an example.

Cortisol plays an important role in lipid and glucose metabolism. Under normal conditions, cortisol peaks in the early morning at the beginning of the behavioral waking period [44–46]. In anticipation of increased activity and energy needs, it promotes controlled fat breakdown through lipolysis and gluconeogenesis in the liver, and acts on the pancreas to decrease insulin and increase glucagon. During overnight work, there is circadian misalignment. The cortisol peak continues to occur early in the morning but this now corresponds to the behavioral sleeping period during which our energy needs are low. The result is inappropriate lipid and glucose metabolism leading to dyslipidemia and a higher risk of type 2 diabetes.

#### Molecular clock and cancer

The master clock in the suprachiasmatic nucleus receives light input to keep our rhythm in-tuned to the light-dark cycle. The master clock also entrains functions at the cellular molecular level. The cellular molecular clock consists of a transcription-translational autoregulatory feedback loop involving clock genes which regulate many physiological processes [47, 48]. For instance, the circadian clock at the molecular level affects the timing of mitosis, cell proliferation, differentiation, DNA repair, and apoptosis. The circadian control of gene expression also affects almost all components of the immune system. Mutations in clock genes are associated with an increased risk of malignancies.

Existing data suggest that clock genes are essential regulators of anti-tumor immunity and the alteration of their expression leads to an immunosuppressive environment that can lead to cancer progression, metastasization, and the development of immune checkpoint escape mechanisms [47, 48]. Altered expression of clock genes related to after-hours work may contribute to an increased risk of cancer. Multiple case-control studies and meta-analyses have demonstrated an association between night-shift-work and breast cancer. One of the largest studies determined the pooled risk to be up to 1.20, with another showing an increased risk in those who engaged in shift-work for over 20 years including during young adulthood [49]. A large case-control study in Spain found that subjects who worked at least 1 year in night-shift-work had slightly higher prostate cancer risk (OR 1.37) [50]. The study similarly demonstrated an increased risk with greater than 28 years of exposure (RRR 1.63).

#### Other disease risks

Numerous other studies demonstrate the association of night-shift-work with an increased risk of various other diseases. Meta-analyses have shown an association between night-shift-work and preterm delivery and early pregnancy loss [51, 52]. Multiple studies have demonstrated an increased risk of cardiovascular disease in night-shift-workers [53]. For instance, a prospective study of over 180,000 nurses followed up over 24 years showed an increased risk of coronary heart disease with increasing years of night-shift-work (CI 1.04–1.24.04.24, *P*=0.004) [54]. The increased coronary heart disease risk is multifactorial owing to night-shift-related lifestyle, physiology, increased inflammatory markers, and hypertension [53]. Finally, mice with mutations in clock-controlled genes have been shown to exhibit markers of accelerated aging [55].

#### Recommendations for mitigating sleep-related performance impairment

The American Academy of Sleep Medicine has published evidence-based recommendations for management of shift-work disorders [56, 57]. Key points from this document include (1) Naps before and during overnight shifts increase alertness during night work and reduce the risk for error without affecting post-shift daytime sleep. (2) Light exposure during the shift and restriction afterward improve performance, alertness, and mood, and may facilitate circadian phase shifts to improve daytime sleep. (3) Melatonin improves daytime sleep quality and duration and contributes to phase shift. (4) Caffeine improves alertness but should be avoided within 6 h of shift end as it may disrupt daytime sleep [58].

Evidence from industries such as aviation demonstrates that structured breaks can be helpful to maintain vigilance, reduce fatigue, and prevent errors [59]. Similar findings have been demonstrated in radiology. A randomized study of radiologists demonstrated a fatigue-related decline in diagnostic accuracy during long reading sessions [60]. Similar declines have also been demonstrated in radiology resident reports with increasing work hours but showed improvement after breaks [61]. Double-staffing or overlapping shifts after-hours to allow for breaks, as well as social interaction, is consequently recommended in literature from various industries, including radiology [5, 16].

Ensuring the environment is conducive to high-quality sleep is also important [5]. Rooms should be dark, cool, quiet, and comfortable. Family and friends should be educated on the importance of daytime sleep, and shift-workers should refrain from taking on responsibilities that will shorten sleeping time.

#### Recommendations for mitigating increased disease risk

Mitigating disease risk associated with night-shift-work should involve a multipronged approach (Table 3). Diet and exercise are known to reduce the risk of cardiovascular disease. A study of factory shift-workers showed that 17 min of high-intensity exercise three times per week significantly improved blood pressure and HbA1c [62]. Similarly, a randomized control study of night-shift-workers showed a significant reduction in atherosclerosis biomarkers after 30 min of brisk walking 3 times per week [63]. Diet is also very important. The Department of Health and Human Services and the American Cancer Society provide general diet recommendations which can be valuable. Additionally, the timing of food intake significantly impacts disease risk. Research suggests limiting nighttime eating, as the body is not optimized for digesting food overnight. Time-restricted eating may help maintain or reset circadian rhythm [44].

**Table 3:** Strategies to mitigate after-hours work challenges

Domain	Strategies	Key points
Sleep/Circadian	Pre-shift and intra-shift naps	Improve alertness
Sleep/Circadian	Light exposure and restriction	Improves performance and aids circadian adaptation; brief, timed bright light therapy during night shifts and blackout curtains after shifts are recommended
Sleep/Circadian	Melatonin	Improves sleep quality and duration
Sleep/Circadian	Caffeine	Improves alertness but may disrupt sleep if taken too late; avoid within 6 h of shift end
Health/Wellness	Exercise	Reduces cardiovascular and metabolic risks; e.g., 30 min of brisk walking 3× per week improves health markers
Health/Wellness	Time-restricted eating	Aligns food intake with circadian rhythms; avoid meals during overnight hours and restrict eating to a daytime window
Operational/Workflow	Workflow interventions	Regular structured breaks, double-staffing/overlapping shifts, and deferring non-urgent imaging can help reduce fatigue and diagnostic errors

## Tools for improving workflow and efficiency

### Artificial intelligences

Integration of AI-based interpretive and non-interpretive tools could further enhance diagnostic accuracy and reduce cognitive load on radiologists working after-hours. AI algorithms could assist with initial readings, prioritizing critical cases for radiologist review, thus reducing the diagnostic burden and speeding up turnaround times. Unfortunately, the vast majority of current AI applications lack sufficient testing in the pediatric population which could limit or degrade their effectiveness for pediatric imaging workflows [64].

### Optimized communication systems

With integration of the Radiological Information Systems (RIS), PACS (Picture Archiving and Communication System) and reporting systems, patient identifiers, and examination information can automatically map into examination reports. There are many potential benefits of report automation to radiologists including improvements in efficiency, accuracy, and fatigue. According to one research study, automatic report population saved radiologists an average of 68 min per day, assuming an average workload of 80 studies [65]. Enhanced communication systems, such as dedicated hotlines or secure messaging platforms, can bridge the gap between emergency clinicians and off-site radiologists during after-hours, enabling quicker decision-making in critical cases. Reading room assistants can similarly facilitate communications between radiologists, technologists, and other clinical staff, as well as off-load other nonclinical tasks [66, 67].

### Workflow standardization

Workflows should aim to reduce unnecessary burden on the emergency-focused overnight staff by deferring non-urgent imaging to better-staffed daytime hours during which subspecialty coverage is more available. Standardized protocols, algorithms, and evidence-based guidelines for pediatric imaging, developed in conjunction with pediatric radiologists, can help streamline processes and decrease imaging frequency without compromising diagnostic quality [68]. For instance, the integration of validated clinical decision tools regarding imaging of pediatric minor head trauma into electronic health records significantly decreases CT utilization with rare reports of clinically significant missed injuries [69, 70].

Additionally, institutional support through cross-campus collaboration can provide critical backup resources. Enhanced education for clinicians, implementation of clinical decision support tools, and system-wide initiatives to align imaging practices with evidence-based guidelines are essential to optimizing imaging utilization [71].

### Remote work options for radiologists

The COVID-19 pandemic dramatically increased the adoption of remote work in academic radiology, initially as a temporary measure but now a long-term component of radiology departments. Benefits include eliminating commutes, improving work-life balance, increasing autonomy, and expanding the pool of potential hires by allowing faculty to work from any location. Remote work has been linked to increased productivity. A study of pediatric neuroradiologists reported faster turnaround times and higher case volumes without compromising accuracy [72]. Disadvantages include decreased visibility of radiologists to referring clinicians, reduced collaboration, limited mentorship opportunities, and potential feelings of isolation among faculty. Inequities arise as some radiology subspecialties (e.g., interventional radiology) require on-site presence, while others (e.g., cross-sectional imaging) can be performed remotely. Additionally, it is important to note that malpractice risks and laws related to the practice of medicine, human resources, and taxation can vary between states and different counties within a given state. This may limit the locations an institution will allow radiologists to practice from remotely. Recommendations include ensuring equitable remote work opportunities, developing strong mentorship programs, incorporating hybrid education formats, and maintaining a critical mass of on-site faculty for visibility and collaboration [72].

## Conclusion

Improving pediatric radiology coverage during after-hours requires a multifaceted approach, addressing staffing models, physiological challenges, workflow, communication barriers, and technological integration. Identifying radiologists with traits associated with better tolerance for circadian shifts and stressors of after-hours work, and designing schedules that improve work-life balance and help to mitigate cognitive impairments related to sleep deprivation may improve job satisfaction, staff retention, and recruitment. Implementation of structured after-hours protocols, investment in artificial intelligence applications and communication technologies, and consideration for radiologists’ well-being are important steps to ensure high-quality patient care in pediatric emergency departments. These changes, supported by evidence and best practices, will help mitigate the unique challenges faced in after-hours pediatric radiology.

## Data Availability

No datasets were generated or analysed during the current study.
